# Incursion of European Bat Lyssavirus 1 (EBLV-1) in Serotine Bats in the United Kingdom

**DOI:** 10.3390/v13101979

**Published:** 2021-10-01

**Authors:** Arran J. Folly, Denise A. Marston, Megan Golding, Shweta Shukla, Rebekah Wilkie, Fabian Z. X. Lean, Alejandro Núñez, Lisa Worledge, James Aegerter, Ashley C. Banyard, Anthony R. Fooks, Nicholas Johnson, Lorraine M. McElhinney

**Affiliations:** 1 Virology Department, Animal and Plant Health Agency, Woodham Lane, Addlestone KT15 3NB, UK; d.marston@surrey.ac.uk (D.A.M.); megan.golding@apha.gov.uk (M.G.); shweta.shukla@apha.gov.uk (S.S.); rebekah.wilkie@apha.gov.uk (R.W.); ashley.banyard@apha.gov.uk (A.C.B.); tony.fooks@apha.gov.uk (A.R.F.); nick.johnson@apha.gov.uk (N.J.); lorraine.mcelhinney@apha.gov.uk (L.M.M.); 2 Pathology and Animal Sciences Department, Animal and Plant Health Agency, Addlestone KT15 3NB, UK; fabian.lean@apha.gov.uk (F.Z.X.L.); alejandro.nunez@apha.gov.uk (A.N.); 3 Bat Conservation Trust, Studio 15 Cloisters House, Cloisters Business Centre, 8 Battersea Park Road, London SW8 4BG, UK; lworledge@bats.org.uk; 4 National Wildlife Management Centre, Animal and Plant Health Agency, Sand Hutton, York YO41 1LZ, UK; james.aegerter@apha.gov.uk

**Keywords:** emerging infectious diseases, bat rabies, *Eptesicus serotinus*, disease surveillance, lyssaviruses, immunohistochemistry

## Abstract

Lyssaviruses are an important genus of zoonotic viruses which cause the disease rabies. The United Kingdom is free of classical rabies (RABV). However, bat rabies due to European bat lyssavirus 2 (EBLV-2), has been detected in Daubenton’s bats (*Myotis daubentonii*) in Great Britain since 1996, including a fatal human case in Scotland in 2002. Across Europe, European bat lyssavirus 1 (EBLV-1) is commonly associated with serotine bats (*Eptesicus serotinus*). Despite the presence of serotine bats across large parts of southern England, EBLV-1 had not previously been detected in this population. However, in 2018, EBLV-1 was detected through passive surveillance in a serotine bat from Dorset, England, using a combination of fluorescent antibody test, reverse transcription-PCR, Sanger sequencing and immunohistochemical analysis. Subsequent EBLV-1 positive serotine bats have been identified in South West England, again through passive surveillance, during 2018, 2019 and 2020. Here, we confirm details of seven cases of EBLV-1 and present similarities in genetic sequence indicating that emergence of EBLV-1 is likely to be recent, potentially associated with the natural movement of bats from the near continent

## 1. Introduction

Naïve animal populations, including those of wild bat species, without previous exposure to infectious novel pathogens may show little resistance to infection or transmission of disease [1,2,3,4]. Consequently, focal cases in vulnerable populations might facilitate incursions, and these, if able to spread may result in epidemic outbreaks or endemic diseases. Where these emergent diseases are zoonotic and become established in animal populations, they can have serious repercussions for human health [5,6,7]. The United Kingdom (UK) is free of classical rabies (RABV) [8]. However, members of the closely related European bat lyssaviruses (EBLV), which cause fatal rabies encephalitis, do circulate in the UK and mainland Europe [9,10]. In the UK, European bat lyssavirus 2 (EBLV-2) is endemic in Daubenton’s bats (*Myotis daubentonii*) and has been detected regularly since 1996 [11,12,13]. The detection of bat lyssaviruses in a wildlife reservoir presents a public health risk, and a fatal human case of EBLV-2 infection was recorded in Scotland in 2002 [14]. Across Europe, European bat lyssavirus 1 (EBLV-1), which clusters into two distinct subgroups occupying overlapping geographic areas (EBLV-1a and EBLV-1b) [15,16], is the most prevalent lyssavirus reported in bat populations [9,17,18], with almost all detections identified in serotine bats (specifically, *Eptesicus serotinus* and the meridional serotine *E. isabellinus*) [19,20]. Fortunately, disease spillover and zoonotic cross-species transmission events are rare [20,21,22,23]. However, within Europe the majority of EBLV cases in humans or animals are due to EBLV-1 in serotine bats [10], and include fatal human infections in Ukraine and most recently France [24,25]. This is likely a consequence of the local disease prevalence and the association of serotine bat roosts with buildings, e.g., homes and offices as well as accommodation for livestock [26]. Definitive mechanisms for the maintenance of EBLV-1 in serotine bat populations have not been established, but the species does form relatively large (n = 20–100 individuals) maternity colonies and a network of infection and or transmission nodes may exist across populations, similar to that seen in Daubenton’s bats which are thought to contribute to the maintenance of EBLV-2 in that system [27,28]. The interconnectivity of these infection nodes may contribute to disease maintenance in bat populations and are also potential pathways supporting natural spread (i.e., emergence into new areas).

Infection with EBLV-1 in humans results in neurological disease and ultimately death, unless prophylactic vaccination and/or rapid post-exposure prophylaxis is provided [29]. The UK has been considered free of EBLV-1, an opinion supported by an ongoing passive surveillance programme that had not detected it over the past three decades. This surveillance is undertaken by the Animal and Plant Health Agency (APHA) in collaboration with a charity the Bat Conservation Trust (BCT), private veterinary surgeons (PVS) and the public [13]. The historical absence of EBLV-1 in UK serotine bats is further corroborated by an active surveillance programme undertaken between 2003–2006 which found no firm evidence of infection [30]. However, in 2018 passive surveillance detected EBLV-1 for the first time in a serotine bat in Dorset, UK, using a combination of diagnostic assays, which specifically detect viral antigens and nucleic acid. Positive tissues have also been subjected to immunochistochemical analysis to confirm the presence and distribution of viral antigens. Subsequent detections of EBLV-1 infection have been made through passive surveillance in 2018, 2019, and 2020, again in serotine bats from Dorset, UK and most recently Somerset, UK. We also detected lyssavirus antigen in a soprano pipistrelle (*Pipistrellus pygmaeus*) recovered close to a number of confirmed EBLV-1 cases, using the fluorescent antibody test (FAT). Here, we confirm details of seven EBLV-1 detections in serotine bats in England and show that similarities in genetic sequence with published isolates suggest that detection in the UK is likely due to natural movement of bats from the near continent and using immunohistochemistry we demonstrate virus distribution to further understand EBLV-1 infection in serotine bats. Combined, these results can inform future surveillance and risk assessments.

## 2. Materials and Methods

### 2.1. UK Lyssavirus Surveillance

The APHA has undertaken passive lyssavirus surveillance in native bats in the UK since 1987 [12,13]. Dead bats are submitted for lyssavirus screening through channels such as BCT and PVSs, or by members of the public. Between 1987 and 2020, 19,887 bats from the UK have been submitted for routine screening. This passive surveillance effort has been sufficient to detect cases of EBLV-2 using FAT and RT-PCR, exclusively in Daubenton’s bats [11,31]. Between 2018 and 2020, 2875 bats were submitted to the APHA for routine lyssavirus testing.

### 2.2. Bat Speciation and Lyssavirus Diagnostic Assays

Bat carcass submissions are initially identified to species level by trained and highly experienced staff using morphology. A brain sample was obtained from each carcass by dissection, using aseptic techniques for downstream analysis. All validated laboratory tests, described below, used for lyssavirus diagnosis, are recommended by the World Health Organisation (WHO) and World Organisation for Animal Health (OIE). To detect lyssavirus antigen using FAT, brain impression smears were created on glass slides and fixed in 100% acetone. These sections were stained with 1/20 FITC Anti-Rabies Monoclonal Globulin (Fujirebio Diagnostics, Malvern, PA, USA) according to the manufacturer’s instructions. A brain impression from a laboratory EBLV-1 infected mouse was used as a positive control. All slides were viewed by two operators under a fluorescence microscope (Leica, Newcastle Upon Tyne, UK) using ultraviolet light to facilitate the detection of viral antigen.

To confirm the FAT diagnosis, total RNA was independently extracted from bat brain tissue using TRIzol (Thermo Fisher Scientific, Loughborough, UK) and resuspended in 10 µL of RNAse free water and diluted to achieve 1 µg/µL. These samples were subjected to several molecular analyses, including a pan-lyssavirus PCR test [32]. Each real-time RT-PCR assay described below was undertaken on an MX-Pro 3000 RT-PCR system (Agilent Technologies, Santa Clara, CA, USA) and data was visualised in MX3000p v4. software (Agilent Technologies, US). Samples were first screened using a pan-lyssavirus SYBR ^®^ RT-PCR designed to detect a 120-base pair (bp) conserved region of the nucleoprotein gene [32]. If samples were positive for the presence of lyssavirus RNA then a further real time RT-PCR was undertaken using a differential TaqMan assay designed to amplify a 145 bp region of the nucleoprotein gene, with three sets of probes each with a unique dye, shown to differentiate between RABV (FAM), EBLV-1 (HEX) and EBLV-2 (Cy5) in a single reaction tube [33]. Lyssavirus positive samples were also screened using a heminested RT-PCR (hnRT-PCR) to produce amplicons sufficient for Sanger sequencing using the primers Jw12 and Jw6 [34]. Amplicons generated by the hnRT-PCR were prepared and sequenced on ABI 3130xl genetic analyser or AB3730 DNA analyser (Applied Biosystems/Life Technologies, Paisley, UK) [13]. Sequences were aligned and quality checked in SeqBuilder Pro (DNAstar, Madison, WI, USA) and the BLAST database was used to identify sequences with high identity. The recovered EBLV-1 sequences from the seven UK serotine bats were aligned with representative EBLV-1a, EBLV-1b and EBLV-2 sequences from Genbank using Muscle (v3.8.31) and a maximum likelihood phylogeny was generated in MEGA X (v10.2.6) with 100 bootstrap replicates.

### 2.3. Immunohistochemical Identification of Viral Antigens from EBLV-1 Infected Bat Tissue

EBLV-1 positive serotine bat specimens were fixed in 10% neutral buffered formalin and decalcified using a 15% (*w*/*v*) EDTA solution adjusted to pH 7.0 with 0.1 M sodium hydroxide (Sigma Aldrich, Gillingham, UK) for seven days and then processed by a routine histology method. A RT-PCR negative serotine bat was included for IHC as a negative control (Supplementary Figure S1). Formalin-fixed paraffin-embedded (FFPE) sections of 4 μm thickness were stained using a Ventana Discovery XT instrument (Ventana Medical Systems, Oro Valley, AR, USA) and the manufacturer’s reagents [35]. Slides were dewaxed using Discovery Wash (Roche, Welwyn Garden City, UK) and epitope retrieval was performed using Cell Conditioning (CC2) solution (pH6 citrate buffer; Roche, UK) for 1 h at 95 °C. Slides were blocked with 5% normal goat, 5% normal swine and 5% normal chicken sera (henceforth referred to as cocktail blocking sera, Vector Laboratories, Burlingame, CA, USA). Primary antibody (mouse monoclonal 5B12 clone at 1/950; MyBioSource, San Diego, CA, USA) or matching isotype matching controls (MsIgG at 1/1000) diluted in cocktail blocking sera were applied on tissue sections and incubated at room temperature (RT) for 2 h. This was followed by the application of a labelled polymer link antibody (OmniMap anti-mouse HRP; Roche, UK) for 32 min at RT with the addition of cocktail blocking sera. Immunolabelling was then visualised with 3,3-diaminobenzidine tetrahydrochorlide (DAB) (OmniMap DAB detection kit; Roche, UK) for 4 min at RT. Slides were counterstained using Hematoxylin (Roche, UK) and bluing reagent (Roche, UK). Finally, before visualisation, slides were rinsed in warm water and detergent to remove the liquid coverslip, followed by dehydration and clearing of sections in absolute alcohol and xylene, and glass coverslips were mounted using Dibutyl Phthalate Xylene (TCS Biosciences, Buckingham, UK).

## 3. Results

### 3.1. EBLV-1 Detection and Sequence Analysis

**Figure 1:**
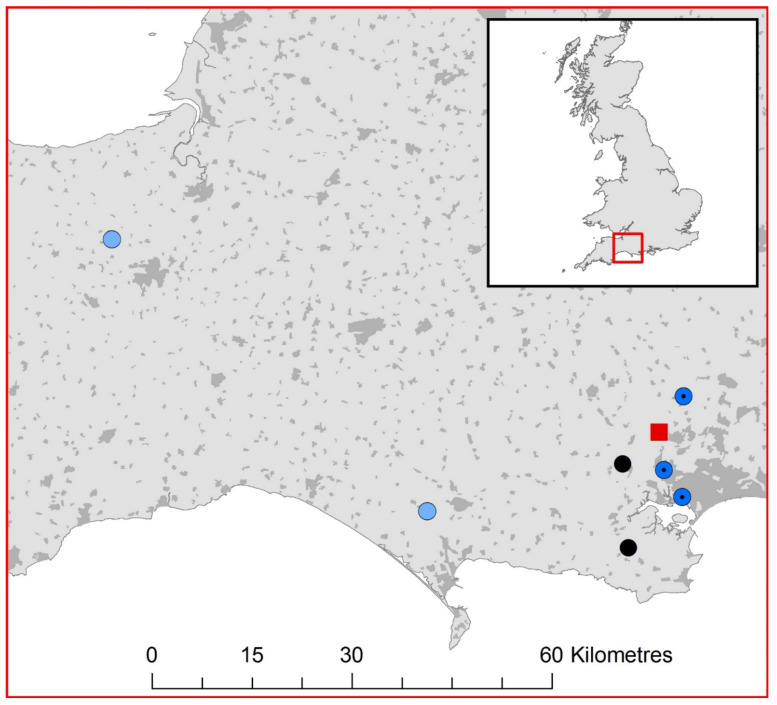
Location of the seven EBLV-1 positive serotine bats (*E. serotinus*) (black circles =2018, filled dark blue circles = 2019, and open light blue circles = 2020) and one lyssavirus FAT positive soprano pipistrelle (*P. pygmaeus*) (red square) in England. The base map highlights urban areas in dark grey.

**Figure 2:**
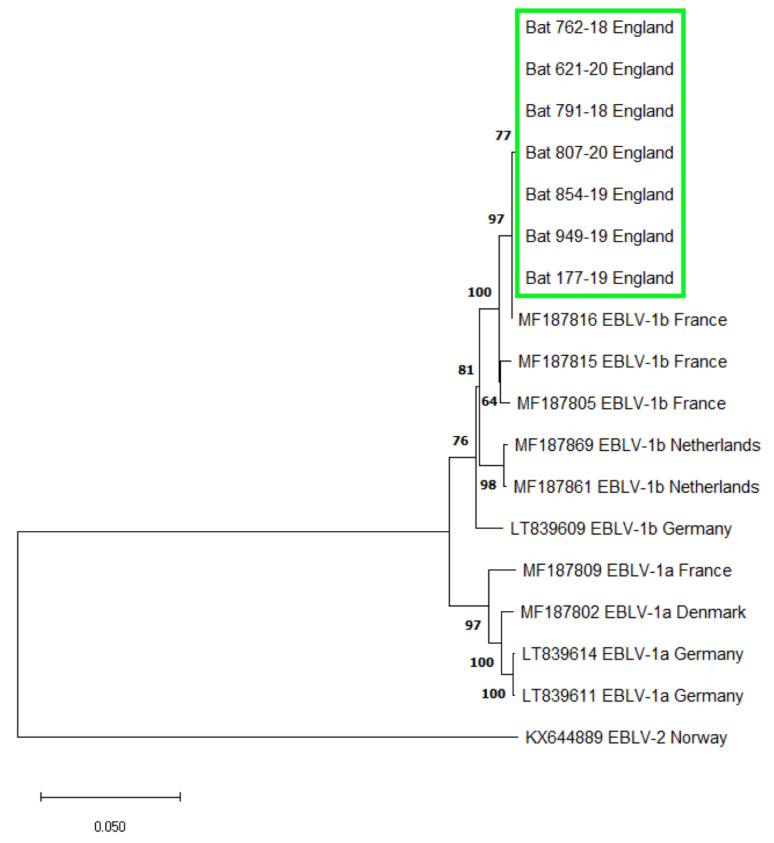
Maximum likelihood phylogeny of seven EBLV-1 detections in the UK (highlighted green), based on 405 bp region of the nucleoprotein gene (nucleotide position 55–459 of the rabies virus genome (Pasteur strain)). The phylogeny includes representative EBLV-1a and EBLV-1b isolates including their Genbank accession number and country of origin. All seven sequences form a well-supported clade with EBLV-1b sequences from France. Node labels represent bootstrap values and scale bar represents substitutions per site.

The APHA passive surveillance scheme for lyssaviruses in bats has tested 229 serotine bats for lyssaviruses since 1987 (1.35% of 17,012 submissions before 2018), although a surprising proportion of these were submitted in the last three years (53 bats between 2018–2020; representing 1.84% of 2875 submissions of all species in that period). Of these 53 serotine bats, seven were found to be positive for the presence of EBLV-1 using FAT, and molecular assays (Figure 1) corresponding to a prevalence of 13% in submitted and screened serotine bats in the UK between 2018 and 2020. The seven lyssavirus positive bats were diagnosed using a suite of diagnostic assays to detect lyssavirus antigen using FAT, and lyssavirus viral RNA using a pan-lyssavirus SYBR real-time RT-PCR. Subsequently, EBLV-1 was confirmed as the lyssavirus species using a differential TaqMan real-time RT-PCR (Table 1) and by sequencing the amplicons generated by a pan-lyssavirus hnRT-PCR. For each positive serotine hnRT-PCR amplification, sanger sequencing produced contigs that were 606 bp in length, as was expected. All seven sequences showed 99.75% similarity to a 405 bp region of the nucleoprotein gene (nucleotide position 55–459 of the rabies virus genome (Pasteur strain, Genbank accession number JX276550)) of an EBLV-1 genome isolated from an adult serotine bat in France (Accession number: MF187816) (Supplementary Table S1) and were 100% identical to each other. Phylogenetic analysis identified all UK EBLV-1 sequences formed a well-supported clade with EBLV-1b isolates (Figure 2). In addition, using FAT, we also detected a lyssavirus in a single juvenile soprano pipistrelle (confirmed by three trained operators). Between 2018 and 2020, 104 soprano pipistrelles were tested, representing 3.62% of 2875 submissions of all species in that period. However, this bat was too degraded through autolysis to enable further molecular (the sample was RT-PCR negative using both the SYBR and Taqman assays described above) or immunohistochemical analysis to corroborate FAT result.

### 3.2. Immunohistochemical Detection of Viral Antigens in EBLV-1 Infected Tissue

**Figure 3:**
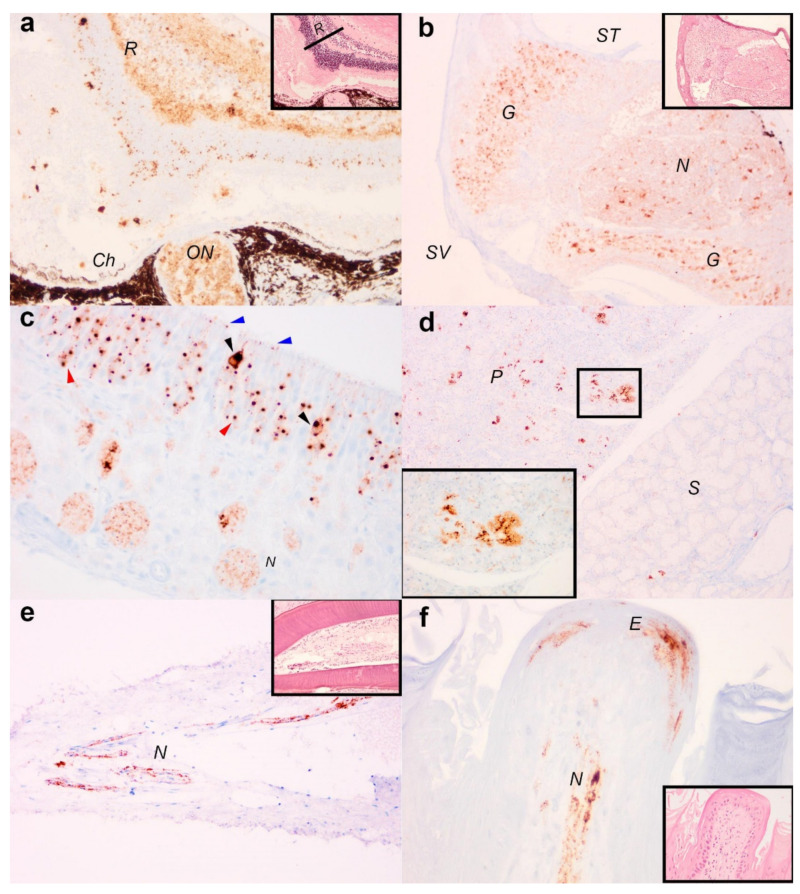
Distribution of EBLV-1 antigens in serotine bats (*E. serotinus*). (**a**) Eye, viral antigens are present in the retina (R) and optic nerve (ON) (images taken at 400×). Ch (choroid plexus). (**b**) Cochlear, viral antigens are present in the spiral ganglion (G) and nerve fibres (N) (images taken at 100×, Scala vestibuli (SV), scala tympani (ST)). (**c**) Olfactory mucosa, viral antigens present in olfactory receptor cells (black arrow heads), basal cells (red arrow heads), cilia (blue arrow heads) and nerve bundles (N) (image taken at 400×). (**d**) Salivary glands, viral antigens are present in the acini (inset, magnified image taken at 400×), with more antigens in parotid gland (P) than the sublingual gland (S) (image taken at 100×). (**e**) Tooth, viral antigens present in nerve fibres within the tooth pulp (images taken at 200×. (**f**) Fungiform papilla of the tongue, viral antigens present in stratified epithelial cells (E) and nerve fibres (N) (images taken at 400×). A selection of H&E stained sections (panels **a**,**b**,**e**,**f**) were included as insets for the purpose of anatomical referencing.

Immunohistochemistry showed all seven serotine bats were positive for viral antigens in the central nervous system including the brain and spinal cord. Where tissues were present for analysis, four bats examined had immunolabelling in the retina and optic nerve (Figure 3a) and the spiral ganglion of the cochlear (Figure 3b). Among the naso-oral structures, viral antigens were present in the olfactory mucosa (6/7 bats), tooth pulp (3/5 bats), salivary glands (4/6 bats) and tongue (4/7 bats). In the olfactory mucosal epithelium, viral antigens were detected in the olfactory receptor cells, basal cells, cilia, and additionally among the nerve bundles within the olfactory submucosa (Figure 3c). In the salivary glands, viral antigens were identified in the acinar epithelial cells (Figure 3d) and also the nervous innervations. In particular, viral antigens were present in greater abundance in the parotid glands than the sublingual glands. Finally, in the oral cavity, viral antigens were present the tooth pulp, in cells with morphology consistent with nerve fibres (Figure 3e). In the tongue, immunolabelling was also present in the muscle spindles and occasionally in the stratified epithelial cells and the submucosa nerve fibres of the fungiform papilla (Figure 3f). Non-specific labelling was not observed in a virus negative bat immunolabelled with primary antibody nor any positive bats upon immunolabelling with isotype controls (Supplementary Figure S1).

## 4. Discussion

Here, we present data that describes the incursion of EBLV-1 in serotine bats in the UK. Our results show that since its first detection in 2018, EBLV-1 was detected in seven serotine bats across three years of passive surveillance. Six cases were clustered within a small area of east Dorset, UK (centred at approximately 50.715 N, −2.086 E) while the seventh case was recovered in Somerset, approximately 85 km west. All seven EBLV-1 detections had identical 405 bp regions of the nucleoprotein gene and were genetically most closely related to a French EBLV-1b isolate. In situ detection of lyssavirus antigens in brain tissue using FAT and IHC detection in FFPE tissue sections from both the central and peripheral nervous systems further demonstrated active viral infection in the bats. Together these results, and the detection of EBLV-1 in juvenile bats which are most likely to have been infected in the UK, suggest that EBLV-1b has emerged in this country. The close similarity of virus found here to that reported in northern France, also in serotine bats, may suggest a natural source of infection through the movement and interaction of the species across the English Channel. As migration or the rates of occasional non-migratory cross-channel movement in serotine bats is very poorly understood [36] it is not clear if these detections represent a single incursion or a more predictable outbreak produced by repeated introductions from the same source.

Given that EBLV-2 has been detected in Daubenton’s bats in the UK and continental Europe [11,31] and that serotine bat populations are present in the UK with a predominantly southerly geographic distribution, the detection of EBLV-1 in serotine bats within the UK is not unexpected and highlights the importance of surveillance programmes to detect the emergence of novel pathogens and understand their potential risk [17]. Not least because some of the most heavily populated regions of England occur within the restricted southerly distribution of serotine bats as well as their almost exclusive synanthropic association with inhabited buildings in England (for their maternity roosts). Consequently, the risk of spillover to humans or pets might be perceived as more significant than the risk of spillover from EBLV-2 in Daubenton’s bat, a species which while more widely distributed, rarely roosts in heated or inhabited buildings, at least in the southern part of the UK, and is generally found living away from people.

The seasonal movements of serotine bats, both in the UK and on the continent, as well as their behaviour away from their maternity roosts are poorly understood. Geneflow between populations of serotine bats on the continent is substantial [36,37,38], although this is not considered a species likely to undertake long-distance movement (especially so for females [37,38]). However, European microsatellite markers have been identified in UK serotine bat populations suggesting movement between the continent and England [36]. While the east Dorset cluster of cases is close to the south coast (all within 16 km of the sea), the closest continental landfall requires a 100 km continuous sea crossing which is hypothesised to be unlikely for serotine bats. However, previous work could not rule-out the possibility of some limited partial migration [36] which may have resulted in the natural movement of EBLV-1 and its detection in England. We would note that passive surveillance undertaken by the APHA, is relatively insensitive and does not screen a sufficient proportion of the UK serotine bat population to conclude that this detection was the initial focal event; it is possible that EBLV-1 was present in UK serotine bats prior to these laboratory confirmed cases, reported here.

In continental serotine bat populations, EBLV-1 seroprevalence can reach up to 40% in multispecies colonies [18] and critically seroprevalence is reportedly higher in the summer months and in adult bats [39]. Consequently, disease maintenance in this system is closely linked to the reproductive behaviour of the host, with aggregations into substantial colonies in summer, a birth pulse of naïve animals and a mechanism promoting transmission between summer colonies such as an autumnal mating behaviour or interaction in shared hibernacula [27,28,40]. Within the UK, the repeated detection of EBLV-2 from single roost sites suggests that populations of Daubenton’s bats maintain EBLV-2 infection, which requires sustained bat-to-bat transmission [41]. Identification of EBLV-2 persistence in these roosts required both passive and active surveillance across multiple years to confirm maintenance in Daubenton’s bats at local and national scales, and it is likely that three years of passive surveillance for EBLV-1 is insufficient to confirm endemic transmission and maintenance within UK serotine bat populations, both of which are key for understanding epidemiology and calculating the potential risk to animal and public health.

The distribution of viral antigens in the tissues of the infected serotine bats reported here demonstrate dissemination of EBLV-1 throughout both the central and peripheral nervous systems, including those within the oral cavity, which is congruent with other lyssaviruses and could be a result of high levels of innervation in these regions [42,43]. European bat lyssavirus transmission is thought to be driven at the individual level by virus shedding during allogrooming, potentially resulting in airborne transmission [44] or more likely via subcutaneous inoculations following a bite from an infected bat [45]. Combined with our detection of viral antigens in tissues of the oral cavity, especially the salivary glands and olfactory mucosa, suggests these sites of virus replication are likely to be important for EBLV release and host-to-host transmission [44,45]. However, the nature of passive surveillance limits the ability to determine the route of infection, or identify the onset of disease, which would be critical in calculating the intrinsic incubation period, or other important epizootilogical rates necessary to predict disease or calculate its risk. Viral nucleic acid and antibodies have been detected in healthy bats, which suggests a variable infection outcome associated with EBLV-1 exposure can occur in serotine bats (e.g., failed infections, sub-clinical infections, and slow disease progression) [46]. Consequently, identifying endemic and persistent infections in serotine bat roosts through passive surveillance remains complex and alternative methods such as environmental sampling (e.g., guano collection or use of hair traps to provide information on colony health status), and active sampling of serotine bats in roosting areas where positive bats have been detected may provide a deeper understanding of EBLV-1 epidemiology [47].

Infection with EBLV-1 causes the disease rabies and can be fatal in bats [12,45,48]. Rabies usually presents itself with neurological impairment which can result in overly aggressive behaviour, motor impairment and poor sensory function [49]. Many of the serotine bat cases described here were submitted after showing signs of neurological impairment (ataxia) or becoming moribund, which when combined with the distribution of viral antigens, shown here, support a viral mediated neurological disease (i.e., rabies). Moreover, our detection of a lyssavirus using FAT (presumed to be EBLV-1 due to close proximity to known EBLV-1 positive roosts, although the sample was negative using RT-PCR methods described above so cannot be confirmed as such) in a juvenile soprano pipistrelle highlights the potential for other species to become infected with bat lyssaviruses [50]. For example, EBLV-1 has been detected using molecular methods in a soprano pipistrelle in Denmark [51]. Indeed, just because EBLV-1 is classically presumed to have a restricted host range ([19], but see also [20,21,22,52,53]), does not mean that people handling bats should not take appropriate prophylactic measures as any species may be a potential risk to human health. Although very rare, fatal human cases caused by EBLV infection have been reported across Europe [14,24,25]. Consequently, the UK advice and guidelines state that anyone who comes into contact with bats should ensure bare skin is covered to prevent bites and those who regularly work with or come into contact with bats should also be vaccinated against rabies, which should provide protection against EBLV-1 and EBLV-2 [28]. Public health guidelines also state that anyone bitten or scratched by a bat should seek medical advice [54]. Where EBLVs are endemic in bat populations across Europe [17,55] there is an increased likelihood of zoonotic spillover events. The continued detection of EBLVs presents a growing yet manageable public health risk [51,54,55,56]. Consequently, an enhanced approach combining passive and active surveillance in bats and using environmental, non-invasive sampling, is required to provide an accurate, ongoing risk assessment in order to effectively mitigate exposure of EBLV in humans and other mammalian species.

## Figures and Tables

**Table 1:** Description, TaqMan RT-PCR result, and case details for seven EBLV-1 positive serotine bats (*E. serotinus*) recovered in the UK between 2018 and 2020.

Bat Species and Unique ID	Date Received	Gender	Age	County	TaqMan RT-PCR Result (Cycle Threshold)	Case Details
*E. serotinus*, Bat 762-18	08/10/2018	Male	Adult	Dorset	Positive EBLV-1 (19.84)	Found dead, possible animal attack.
*E. serotinus*, Bat 791-18	17/10/2018	Female	Juvenile	Dorset	Positive EBLV-1 (22.63)	Died in captivity. Possible animal attack, found grounded near roost.
*E. serotinus*, Bat 177-19	25/05/2019	Male	Adult	Dorset	Positive EBLV-1 (19.37)	Died in captivity. Flew into window and fell to the ground.
*E. serotinus*, Bat 854-19	30/08/2019	Male	Juvenile	Dorset	Positive EBLV-1 (23.99)	Euthanised. Recovered from a public space. Possible animal attack.
*E. serotinus*, Bat 949-19	13/09/2019	Male	Juvenile	Dorset	Positive EBLV-1 (18.54)	Euthanised. Found on public highway path. Signs of animal attack.
*E. serotinus*, Bat 621-20	14/09/2020	Female	Adult	Dorset	Positive EBLV-1 (21.61)	Euthanised. Found grounded.
*E. serotinus*, Bat 807-20	23/12/2020	Female	Adult	Somerset	Positive EBLV-1 (19.55)	Euthanised. Recovered crawling on public road, unable to fly.

## Data Availability

The sequence, which relates to a 405 bp region of the EBLV-1 nucleoprotein gene, recovered from all seven serotine bats has been submitted to Genbank and is awaiting an accession number. Submission number is 2491778.

## Supplementary Materials

The following are available online at https://www.mdpi.com/article/10.3390/v13101979/s1, Figure S1: Immunohistochemistry control tissues of lyssavirus RT-PCR negative serotine bats (*Eptesicus serotinus*), Table S1: Sanger derived sequences for the seven EBLV-1 infected serotine bats (*E. serotinus*) detected in the UK between 2018 and 2021.
