# Supplementary material for: Incursion of European Bat Lyssavirus 1 (EBLV-1) in Serotine Bats in the United Kingdom
Source: Viruses. 2021 Oct 1;13(10):1979. doi: 10.3390/v13101979 (PMC8536961; doi:10.3390/v13101979)
Supplement: Supplementary file 1 [file viruses-13-01979-s001.zip › viruses-1367163-supplementary.pdf]

Supplementary files

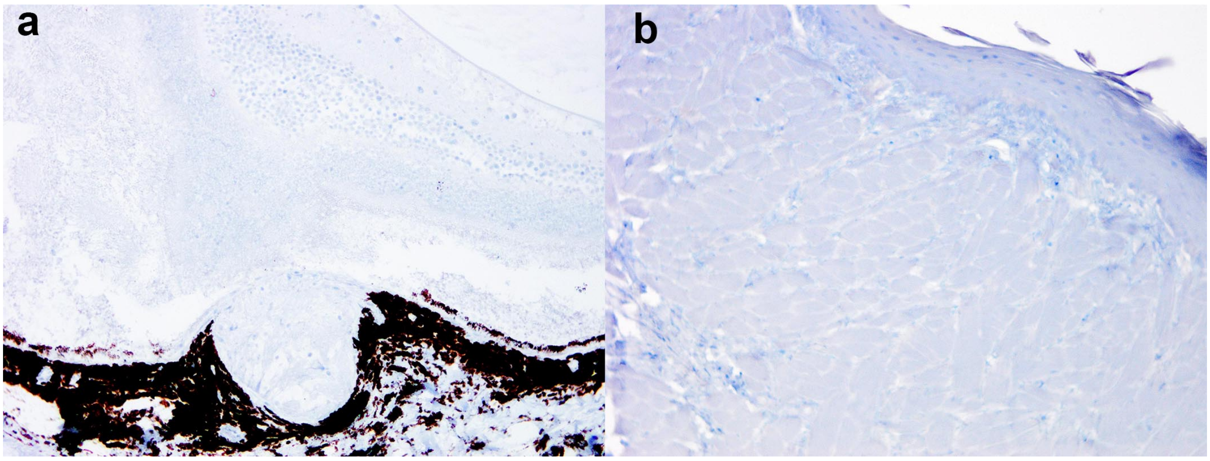

Figure S1. Immunohistochemistry control tissues of lyssavirus RT-PCR negative serotine bats (*Eptesicus serotinus*). (a) Serial sectioned of eye as per Figure 2.a with mouse IgG control. (b) Section of tongue from a lyssavirus negative serotine bat stained with 512B lyssavirus IHC. No non-specific labelling was detected in either control stained sections. Images taken at 200x.

Table S1. Sanger derived sequences for the seven EBLV-1 infected serotine bats (*E. serotinus*) detected in the UK between 2018 and 2021. Sequence for 762-18 deposited on Genbank accession number: MZ821029.

APHA bat ID	Location	Sequence
762-18	Dorset	ATGGATGTTAACAAGGTTGTTTTTAAGGTCCATAATCAGTTGGTCTCGGTGAAACCTGAGGTGATTTCTGATCAGTATGAGTACAAATACCCTGCCATTA<br>AAGACAAGAAGAAACCGAGCATCACTCTCGGAAAAGATCCTGATTTGAAAACCGCCTACAAGTCTATCTTGTCAGGGATGAATGCTGCTAAATTGGACCC<br>AGATGACGTCTGCTCCTATTTAGCTGGAGCCATGGTCTTGTTTGAGGGCATATGCCCAGAAGATTGGACTAGTTACGGAATCAACATTGCAAAGAAAGGT<br>GACAAGATCACACCTGCTACGTTAGTGGACATCAATCGGACGAACACTGAGGGCAACTGGGCTCAAACAGGAGGTCAAGATCTCACTCGGGACCCTACGA<br>CACCT
791-18	Dorset	ATGGATGTTAACAAGGTTGTTTTTAAGGTCCATAATCAGTTGGTCTCGGTGAAACCTGAGGTGATTTCTGATCAGTATGAGTACAAATACCCTGCCATTA<br>AAGACAAGAAGAAACCGAGCATCACTCTCGGAAAAGATCCTGATTTGAAAACCGCCTACAAGTCTATCTTGTCAGGGATGAATGCTGCTAAATTGGACCC<br>AGATGACGTCTGCTCCTATTTAGCTGGAGCCATGGTCTTGTTTGAGGGCATATGCCCAGAAGATTGGACTAGTTACGGAATCAACATTGCAAAGAAAGGT<br>GACAAGATCACACCTGCTACGTTAGTGGACATCAATCGGACGAACACTGAGGGCAACTGGGCTCAAACAGGAGGTCAAGATCTCACTCGGGACCCTACGA<br>CACCT
177-19	Dorset	ATGGATGTTAACAAGGTTGTTTTTAAGGTCCATAATCAGTTGGTCTCGGTGAAACCTGAGGTGATTTCTGATCAGTATGAGTACAAATACCCTGCCATTA<br>AAGACAAGAAGAAACCGAGCATCACTCTCGGAAAAGATCCTGATTTGAAAACCGCCTACAAGTCTATCTTGTCAGGGATGAATGCTGCTAAATTGGACCC<br>AGATGACGTCTGCTCCTATTTAGCTGGAGCCATGGTCTTGTTTGAGGGCATATGCCCAGAAGATTGGACTAGTTACGGAATCAACATTGCAAAGAAAGGT<br>GACAAGATCACACCTGCTACGTTAGTGGACATCAATCGGACGAACACTGAGGGCAACTGGGCTCAAACAGGAGGTCAAGATCTCACTCGGGACCCTACGA<br>CACCT
854-19	Dorset	ATGGATGTTAACAAGGTTGTTTTTAAGGTCCATAATCAGTTGGTCTCGGTGAAACCTGAGGTGATTTCTGATCAGTATGAGTACAAATACCCTGCCATTA<br>AAGACAAGAAGAAACCGAGCATCACTCTCGGAAAAGATCCTGATTTGAAAACCGCCTACAAGTCTATCTTGTCAGGGATGAATGCTGCTAAATTGGACCC<br>AGATGACGTCTGCTCCTATTTAGCTGGAGCCATGGTCTTGTTTGAGGGCATATGCCCAGAAGATTGGACTAGTTACGGAATCAACATTGCAAAGAAAGGT<br>GACAAGATCACACCTGCTACGTTAGTGGACATCAATCGGACGAACACTGAGGGCAACTGGGCTCAAACAGGAGGTCAAGATCTCACTCGGGACCCTACGA<br>CACCT
949-19	Dorset	ATGGATGTTAACAAGGTTGTTTTTAAGGTCCATAATCAGTTGGTCTCGGTGAAACCTGAGGTGATTTCTGATCAGTATGAGTACAAATACCCTGCCATTA<br>AAGACAAGAAGAAACCGAGCATCACTCTCGGAAAAGATCCTGATTTGAAAACCGCCTACAAGTCTATCTTGTCAGGGATGAATGCTGCTAAATTGGACCC<br>AGATGACGTCTGCTCCTATTTAGCTGGAGCCATGGTCTTGTTTGAGGGCATATGCCCAGAAGATTGGACTAGTTACGGAATCAACATTGCAAAGAAAGGT<br>GACAAGATCACACCTGCTACGTTAGTGGACATCAATCGGACGAACACTGAGGGCAACTGGGCTCAAACAGGAGGTCAAGATCTCACTCGGGACCCTACGA<br>CACCT
621-20	Dorset	ATGGATGTTAACAAGGTTGTTTTTAAGGTCCATAATCAGTTGGTCTCGGTGAAACCTGAGGTGATTTCTGATCAGTATGAGTACAAATACCCTGCCATTA<br>AAGACAAGAAGAAACCGAGCATCACTCTCGGAAAAGATCCTGATTTGAAAACCGCCTACAAGTCTATCTTGTCAGGGATGAATGCTGCTAAATTGGACCC<br>AGATGACGTCTGCTCCTATTTAGCTGGAGCCATGGTCTTGTTTGAGGGCATATGCCCAGAAGATTGGACTAGTTACGGAATCAACATTGCAAAGAAAGGT<br>GACAAGATCACACCTGCTACGTTAGTGGACATCAATCGGACGAACACTGAGGGCAACTGGGCTCAAACAGGAGGTCAAGATCTCACTCGGGACCCTACGA<br>CACCT
807-20	Somerset	ATGGATGTTAACAAGGTTGTTTTTAAGGTCCATAATCAGTTGGTCTCGGTGAAACCTGAGGTGATTTCTGATCAGTATGAGTACAAATACCCTGCCATTA<br>AAGACAAGAAGAAACCGAGCATCACTCTCGGAAAAGATCCTGATTTGAAAACCGCCTACAAGTCTATCTTGTCAGGGATGAATGCTGCTAAATTGGACCC<br>AGATGACGTCTGCTCCTATTTAGCTGGAGCCATGGTCTTGTTTGAGGGCATATGCCCAGAAGATTGGACTAGTTACGGAATCAACATTGCAAAGAAAGGT<br>GACAAGATCACACCTGCTACGTTAGTGGACATCAATCGGACGAACACTGAGGGCAACTGGGCTCAAACAGGAGGTCAAGATCTCACTCGGGACCCTACGA<br>CACCT
